# Cytokine signatures associated with persistent antibody responses to multiple *Plasmodium vivax* blood-stage antigens

**DOI:** 10.3389/fimmu.2026.1899417

**Published:** 2026-08-31

**Authors:** Sâmick L. Moreira-Nascimento, Maria Fernanda A. Nascimento, Gabriela M. Fernandes, Sofia L. Afonso, Victor H. G. Gunderman, Bárbara A. Rodrigues, Stefanie C. P. Lopes, Camila Fabbri, Marcus V. G. Lacerda, Gregório G. Almeida, Rhea J. Longley, Ivo Mueller, Lis R. D. V. Antonelli, Francis B. Ntumngia, John H. Adams, Andréa Teixeira-Carvalho, Luzia H. Carvalho, Flora S. Kano

**Affiliations:** 1 Fundação Oswaldo Cruz, Fiocruz, Instituto René Rachou, Fiocruz Minas, Belo Horizonte, Minas Gerais, Brazil; 2 Fundação Oswaldo Cruz, Fiocruz, Instituto Leônidas e Maria Deane, Fiocruz Amazônia, Manaus, Amazonas, Brazil; 3 Universidade Federal do Amazonas, Manaus, Amazonas, Brazil; 4 Fundação de Medicina Tropical Dr Heitor Vieira Dourado, Manaus, Brazil; 5 Universidade Federal de Minas Gerais, Centro de Tecnologia de Vacinas, Belo Horizonte, Minas Gerais, Brazil; 6 The Walter and Eliza Hall Institute of Medical Research, Parkville, Victoria, Australia; 7 Department of Medical Biology, The University of Melbourne, Melbourne, VIC, Australia; 8 Mahidol Vivax Research Unit, Faculty of Tropical Medicine, Mahidol University, Bangkok, Thailand; 9 Center for Global Health and Infectious Diseases Research, College of Public Health, University of South Florida, Tampa, FL, United States

**Keywords:** blood-stage antigens, cytokines, Duffy binding protein II, long-term humoral immunity, malaria vaccine, *Plasmodium vivax*

## Abstract

**Introduction:**

Long-term humoral immunity to *Plasmodium vivax* remains poorly understood, particularly the mechanisms sustaining persistent antibody responses to Duffy Binding Protein region II (DBPII), a key vaccine candidate. Here, a 15-year follow-up study of Amazonian malaria-exposed individuals investigated major cytokine pathways associated with sustained IgG responses to DBPII-related antigens, including DEKnull-2, an engineered immunogen targeting conserved DBPII epitopes.

**Methods:**

As a proof-of-concept, this study was designed to compare two well-defined immunological phenotypes among *P. vivax-*exposed individuals: (i) Persistent Responders (PR, n = 11), who developed a long-lasting strain-transcending anti-DBPII immune response, defined here as sustained antibody responses to both DBPII and DEKnull-2, together with detectable binding-inhibitory antibody activity (BIAbs), throughout the follow-up period; and (ii) Non-Responders (NR; n = 14), who showed no detectable antibody response or BIAbs activity during follow-up. Cytokine responses were measured in supernatants of PBMC cultures stimulated with DBPII or DEKnull-2 by cytometric bead array (CBA), and IgG breadth was assessed using a panel of 16 P*. vivax* blood-stage antigens using Bio-Plex assay.

**Results:**

Regardless of the antigen used for stimulation, *P. vivax*-exposed individuals (NR and PR) exhibited lower IL-6 and TNF levels than malaria-naïve non-exposed controls (NE, n = 14). Notably, the PR subgroup displayed a distinct profile characterized by a coordinated increase across inflammatory and anti-inflammatory cytokines. Even without antigen-specific stimulation, the PR group showed a distinct cytokine profile compared with NE controls, suggesting that host-intrinsic factors may influence DBPII-specific immune responses. Finally, PR (but not NR) displayed broader long-lasting antibody responses to multiple *P. vivax* blood-stage antigens, even in the absence of frequent reinfection-induced boosting.

**Conclusion:**

Overall, these findings suggest that the PR profile is associated with a distinct and balanced cytokine profile. Our hypothesis-generating findings may improve our understanding of immune responses to *P. vivax* blood-stage antigens and inform future vaccine development.

## 1 Introduction

Malaria caused by *Plasmodium vivax* remains a major public health challenge, partly due to the biological complexity of the parasite and the lack of an effective vaccine (1). In this context, the identification of parasite antigens capable of inducing protective immune responses represents a critical step for vaccine development. Multi-antigen vaccine strategies have been increasingly explored, highlighting the importance of selecting targets able to elicit broad and functionally relevant immune responses (2, 3).

Among *P. vivax* blood-stage antigens, region II of the Duffy-binding protein (DBPII) is considered one of the leading vaccine candidates (4). DBPII mediates parasite invasion through binding to the human atypical chemokine receptor 1 (ACKR1), also known as Duffy antigen receptor for chemokines (DARC), expressed on reticulocytes (5, 6). This interaction represents the only well-characterized pathway leading to formation of the irreversible junction required for reticulocyte invasion (7, 8). However, recent reports of *P. vivax* transmission among DARC-negative individuals in Africa (9) highlight additional biological complexities that may limit the global applicability of vaccines based on DBPII. Furthermore, although DBPII-based vaccines have shown some degree of protection (4), the high polymorphism of DBPII promotes strain-specific antibody responses (10–12). To overcome these limitations, novel engineered DBPII immunogens have been developed (13–15). Among these, the surface-engineered DEKnull-2, designed to redirect antibody responses toward conserved DBPII epitopes, enhanced induction of broadly neutralizing antibodies and showed high serological reactivity in individuals with long-term malaria exposure (16). Building on these observations, we previously identified in the Brazilian Amazon, a subgroup of Persistent Responders (PR) who maintained long-lasting, strain-transcending antibody responses to DBPII (DEKnull-2), together with detectable binding-inhibitory antibody activity (BIAbs) against the DBPII–DARC interaction (17, 18). Importantly, antibodies that block the DBPII-DARC interaction have been associated with clinical immunity to *P. vivax* malaria (19), including in rural Amazonians (20).

Despite the central role of antibodies in protective immunity against malaria, the mechanisms that sustain long-lasting humoral responses in naturally exposed individuals remain incompletely understood (21, 22). The generation and maintenance of durable antibody responses depend on coordinated interactions between innate and adaptive immune pathways, in which cytokines act as key regulators of immune activation, cellular differentiation, and memory maintenance (23, 24). Thus, coordinated inflammatory and regulatory patterns involving cytokines and their association with different T helper cell profiles, may influence the quality and stability of antibody responses against *P. vivax* blood-stage antigens (25–27).

Given the central role of cytokines in shaping adaptive immune responses, in this proof-of-concept study, we hypothesized that coordinated cytokine signatures may support the development and maintenance of persistent inhibitory antibody responses to *P. vivax* blood-stage antigens. Therefore, the present study aimed to characterize cytokine profiles associated with long-lasting antibody responses to DBPII-based antigens (DBPII-Sal1 and DEKnull-2) and to determine whether these immune signatures are linked to broader IgG reactivity against multiple *P. vivax* blood-stage vaccine candidate antigens.

## 2 Materials and methods

### 2.1 Area and study population

**Figure 1:**
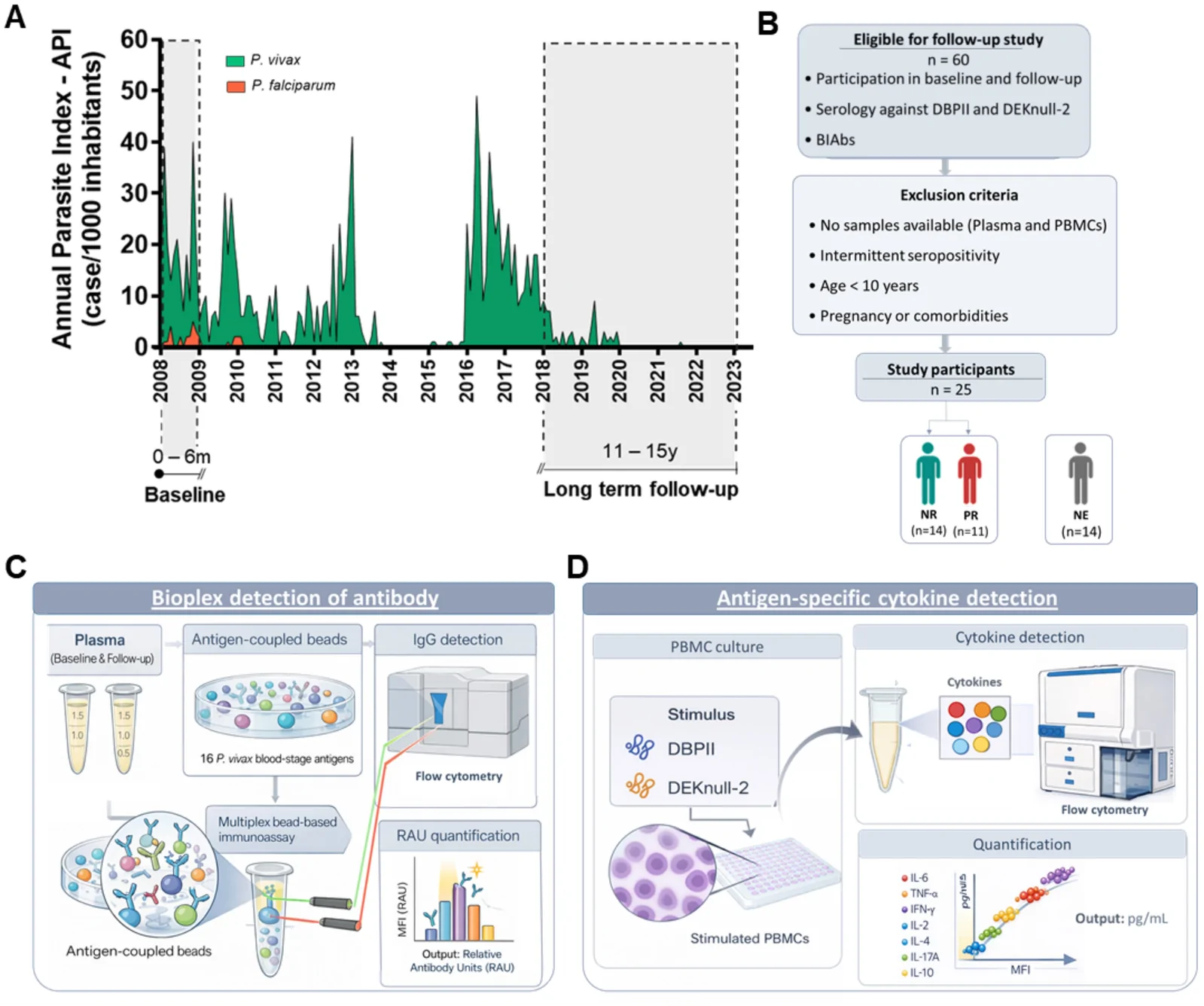
Monthly-time series of microscopically confirmed malaria cases in the study area and the rationale for the study design. **(A)** Malaria cases by *Plasmodium* species represented by the Annual Parasite Index (API; number of cases per 1,000 inhabitants). Periods corresponding to baseline (within 6 months of study onset) and long-term follow-up (> 10 years) are highlighted in gray (dotted rectangles). Epidemiological data were obtained from the National Malaria Epidemiological Surveillance Information System (SIVEP-Malaria), with diagnosis by light microscopy for *P. falciparum* (red) and *P. vivax* (green). **(B)** Participant selection flowchart. The final cohort included 25 malaria-exposed individuals, classified as non-responders (NR, n = 14) and persistent responders (PR, n = 11), as well as 14 malaria-naïve individuals from a non-endemic area (NE) as controls. **(C)** Plasma IgG levels against 16 P*. vivax* blood-stage antigens were measured at baseline and follow-up using a multiplex assay (Bio-Plex). Results were expressed as relative antibody units (RAU), derived from the mean fluorescence intensity (MFI) values for each antigen. **(D)** At the end of the follow-up period, cytokines conventionally associated with Th1-, Th2-, and Th17-type immune profiles, were assessed in the supernatant of PBMCs stimulated with DBPII or DEKnull-2 using a cytometric bead array (CBA). Cytokine concentrations were calculated from MFI values and expressed in pg/mL. The schematic elements illustrating the experimental workflow were created with the assistance of AI-based tools (Google Gemini) for illustrative purposes only.

The study was conducted in the Rio Pardo agricultural settlement (1°46′S-1°54′S, 60°22′W-60°10′W), located in the Presidente Figueiredo municipality, in the northeast of the state of Amazonas in the Brazilian Amazon region (**Supplementary Figure 1**). In this area, malaria transmission is considered hypo- to mesoendemic, and most residents are native to the Amazon region, with a history of long-term exposure to relatively low intensity of malaria transmission (28, 29). The population primarily relies on subsistence agriculture and fishing (28). Throughout the study period, malaria cases fluctuated in the study area, with alternating periods of higher and lower seasonal transmission (**Figure 1A**). The marked decline in malaria transmission during the later years of the follow-up period was consistent with the success of governmental malaria control efforts (30). Since 2010, *P. vivax* has been responsible for all clinical cases of malaria within our study region.

### 2.2 Study design and participants

A population-based open-cohort study was initiated in our study community in November 2008, with long-term follow-up carried out 11 to 15 years later (**Figure 1A**), as previously described (28, 31). Briefly, each survey included: (i) structured questionnaire-based interviews to obtain demographic, epidemiological, and parasitological data; (ii) physical examinations with standardized body temperature measurements; (iii) collection of venous blood samples (5 mL in EDTA for plasma and whole DNA; 9 mL in heparin for PBMC); (iv) preparation of Giemsa-stained thick blood smears for microscopic malaria diagnosis; and (v) use of genomic DNA for species-specific PCR assays targeting different plasmodial targets (32). The geographic coordinates of each household were recorded using a handheld 12-channel Global Positioning System (GPS) device (Garmin 12XL, Olathe, KS, USA) with a positional accuracy of approximately 15 meters.

Given the observational nature of the study and the open-cohort design, the study population comprised a convenience sample that included all eligible participants available during the study period, rather than a selected subset. Individual-level data were retrieved from an EpiData database (http://www.epidata.dk), which was accessed between June 27, 2025, and January 30, 2026. In the database, individual data were de-identified, and participants were coded with no personal identifiers linked to the research data. The methodological strategy was designed to include individuals whose samples were serologically screened for reactivity to DBPII/DEKnull-2 at the time of the baseline and after 10 years of follow-up (11–15 years later) (**Figure 1A**). A total of 60 out of 272 original cohort participants met these criteria (**Supplementary Figure 2**). Additional exclusion criteria included: (i) unavailable consecutive samples (plasma or PBMC); (ii) intermittent seropositivity to DBPII or DEKnull-2; (iii) children under 10 years of age; (iv) pregnancy; and (v) other traceable comorbidities. After applying these criteria, 25 out of 60 individuals were eligible for the present study (**Figure 1B**; **Supplementary Figure 2**). A participant selection flow diagram showing the participants retained and excluded from the cohort were statistically comparable to the original cohort with respect to demographic, epidemiological and parasitological characteristics (**Supplementary Table 1**). According to their antibody response profile, the 25 eligible participants were classified into two subgroups: (i) Persistent Responders (PR; n = 11), who exhibited a long-lasting strain-transcending anti-DBPII immune response, defined here as sustained ELISA-detected antibody responses to both DBPII and DEKnull-2, together with functional antibodies capable of inhibiting the DBPII–DARC interaction (i.e., binding-inhibitory antibodies, BIAbs); and (ii) Non-Responders (NR; n = 14), who showed no detectable antibody response or BIAbs activity during the follow-up (**Figure 1B**). Regarding place of residence, all PR individuals (11/11, 100%) lived in the riverine area, where exposure to mosquito bites is higher than in the non-riverine area (28, 33). In comparison, 50% of the NR individuals (7/14) also resided in the riverine area. The number of previous self-reported malaria episodes was similar between groups (median of 7 and 4 episodes in PR and NR individuals, respectively). Although low levels of *P. falciparum* transmission were detected at the beginning of the cohort (Period I, **Figure 1A**), all malaria infections detected among PR and NR were caused by *P. vivax*, as determined by PCR-based protocols (32). As non-exposed controls (NE), an age-matched malaria-naïve subgroup (n = 14) was included, composed of individuals from a Brazilian non-endemic region, who had never been exposed to malaria transmission.

### 2.3 Plasma and peripheral blood mononuclear cell isolation

Peripheral blood samples were used to obtain plasma and PBMCs. Plasma samples were obtained by centrifugation (500 × g, 10 min, room temperature) and stored at -20 °C until use. PBMCs were isolated by density-gradient centrifugation using Ficoll-Paque (Sigma-Aldrich) following standard protocols (29). Purified PBMCs were resuspended in fetal bovine serum (FBS; Gibco) and their concentration adjusted to 5×10^6^ or 1×10^7^ cells/mL in FBS containing 10% dimethyl sulfoxide (DMSO; Sigma-Aldrich). Cell suspensions were frozen in isopropanol freezing containers (Nalgene) at -80 °C for 24h and then transferred to liquid nitrogen containers until use.

### 2.4 DBPII-related antigens and serological assays

Recombinant DBPII-Sal1, corresponding to amino acids 243–573 of the Duffy binding protein region II (DBPII) of the *P. vivax* Sal1 reference strain, and the surface-engineered immunogen DEKnull-2 were produced as 39-kDa 6×His-tag fusion proteins and refolded according to previously published protocols (34, 35).

IgG antibodies against *P. vivax* recombinant proteins were measured by conventional ELISA, as previously described (34). Serum samples were tested at a 1:100 dilution, and recombinant antigens were coated at 3 μg/mL. The results were expressed as ELISA reactivity index (RI), calculated as the ratio of the mean optical density (OD at 492 nm) of each sample to the mean OD plus three standard deviations of negative control plasma samples from 30 individuals living in a non-endemic area for malaria (Belo Horizonte, Minas Gerais, Brazil) and who have never been exposed to malaria transmission (malaria-naïve individuals). RI values > 1.0 were considered seropositive.

The functional activity of anti-DBPII antibodies (binding-inhibitory antibodies, BIAbs) was assessed using a standard COS-7 cell assay (33). Plasma samples were tested for inhibition of DBPII-erythrocyte binding at 1:40 dilution. Binding was quantified by microscopic counting of rosettes in 10 to 20 fields (×200 magnification), and percent inhibition was calculated relative to negative control plasma. A pool of *P. vivax* immune sera and naïve sera served as positive and negative controls, respectively. Samples showing >50% inhibition were classified as BIAbs-positive.

### 2.5 Bio-Plex detection of antibodies against a panel of *P. vivax* blood-stage antigens

The breadth of antibody responses was assessed using a customized *P. vivax* multiplex panel on the Bio-Plex^®^ 200 platform (Bio-Rad) (**Figure 1C**), adapted from Longley et al. (36). The panel comprised 16 recombinant *P. vivax* blood-stage antigens (**Supplementary Table 2**): RBP2b (N- and C-termini), RBP2a, DBPII-Brz2, EBP2, AMA1, RIPR, MSP1-19, MSP3a, MSP5, MSP8, PTEX150, RAMA, S16, and Pv-Fam-a (34-end and 61-end). Each antigen was individually coupled to a distinct magnetic bead region, and all bead sets were pooled in each reaction well, allowing the simultaneous detection of IgG responses to each antigen (**Figure 1C**).

Briefly, antigen-coupled beads (50µL) were incubated with diluted plasma samples (1:100) for 30 min, followed by incubation with phycoerythrin-conjugated anti-human IgG (1:100) for 15 min. Plasma samples obtained during baseline and long-term follow-up of the cohort study were used. The median fluorescence intensity (MFI) values for each antigen were acquired using the Bio-Plex 200 system. A standard curve composed of hyperimmune control plasma, prepared through serial dilutions (1:50 to 1:25,600), was included on each plate for quality control, normalization, and monitoring of inter-assay variability. MFI values were subsequently converted into relative antibody units (RAU) using each plate-specific standard curve. Seropositivity thresholds were defined based on malaria-naïve controls. Blank wells were included to ensure assay reliability.

### 2.6 Cytokine detection following *in vitro* stimulation

Cytokine profiles were assessed in PBMCs isolated from malaria-exposed (PR and NR) and non-exposed (NE) individuals following *in vitro* stimulation with recombinant DBPII and DEKnull-2 (**Figure 1D**). PBMCs were cultured with DBPII-Sal1 or DEKnull-2 (5 μg/mL) for six days under standard culture conditions (37). Unstimulated cultures served as controls, and all conditions were tested in duplicate (**Supplementary Data**).

Culture supernatants were collected, and cytokine levels were analyzed using BD™ Cytometric Bead Array (CBA) Human Th1/Th2/Th17 Kit (BD Biosciences, San Diego, CA, USA; Cat. No. 560484), following the manufacturer’s instructions. The panel included IL-6, TNF, IFN-γ, IL-2, IL-4, IL-17A, and IL-10. Samples were acquired on a BD FACSVerse™ cytometer, and data analysis was performed using FCAP Array v3.0 software. Cytokine concentrations from antigen-stimulated cultures were analyzed as raw values, without subtraction of background levels from unstimulated cultures, as baseline cytokine production may vary among study groups. This approach was applied consistently across all experimental conditions and subgroups. A detailed standard operating procedure (SOP) describing the PBMC culture and antigen-specific stimulation protocol, including reagents, equipment, procedures, quality-control measures, and critical notes, is provided as **Supplementary Material** (**Supplementary Data**).

### 2.7 Data analysis

Graphs and statistical analyses were performed using GraphPad Prism version 9 (www.graphpad.com) and R software (version 4.4.1). Data distribution was assessed using the Shapiro-Wilk test. Comparisons of proportions were performed using the chi-square (χ²) test or Fisher’s exact test, as appropriate. Depending on data distribution (normal or non-normal), differences between subgroups for continuous variables were assessed using one-way ANOVA with appropriate *post hoc* tests, or Mann-Whitney and Kruskal-Wallis test followed by Dunn’s multiple-comparison test. A Bonferroni correction was applied for multiple comparisons to reduce the risk of type I errors arising from repeated statistical testing. Paired longitudinal comparisons were conducted using paired t tests or Wilcoxon signed-rank tests, as appropriate. Outliers were assessed using statistical methods appropriate to the data distribution. Statistical significance was defined as p < 0.05. To evaluate the magnitude of the difference between groups, pairwise effect sizes were estimated using Cliff’s delta (δ) for comparisons, with 95% bootstrap confidence intervals (38). Effect sizes were classified as negligible (|δ| < 0.147), small (0.147–0.329), medium (0.330–0.474), or large (|δ| ≥ 0.475).

Principal component analysis (PCA) was used as an unsupervised descriptive approach to evaluate coordinated immune response patterns using Z-score-normalized cytokine responses. Analyses were conducted separately for each stimulus (DBPII and DEKnull-2) in R (prcomp function), and the two principal components were used for visualization. Radial signature analysis was performed to provide a descriptive assessment of integrated cytokine profiles. For each cytokine and stimulus, the global median across all participants was used as the cut-off to classify individual responses as above or below the threshold. Results were expressed as the proportion (%) of participants in each subgroup with cytokine levels above the global median, allowing group-independent comparison of the proportion of individuals with higher cytokine responses while minimizing the influence of extreme values. All data necessary to replicate the findings of our study are openly available (**Supplementary Table 3**).

## 3 Results

### 3.1 Cytokine profile associated with DBPII and DEKnull-2 responders

**Figure 2:**
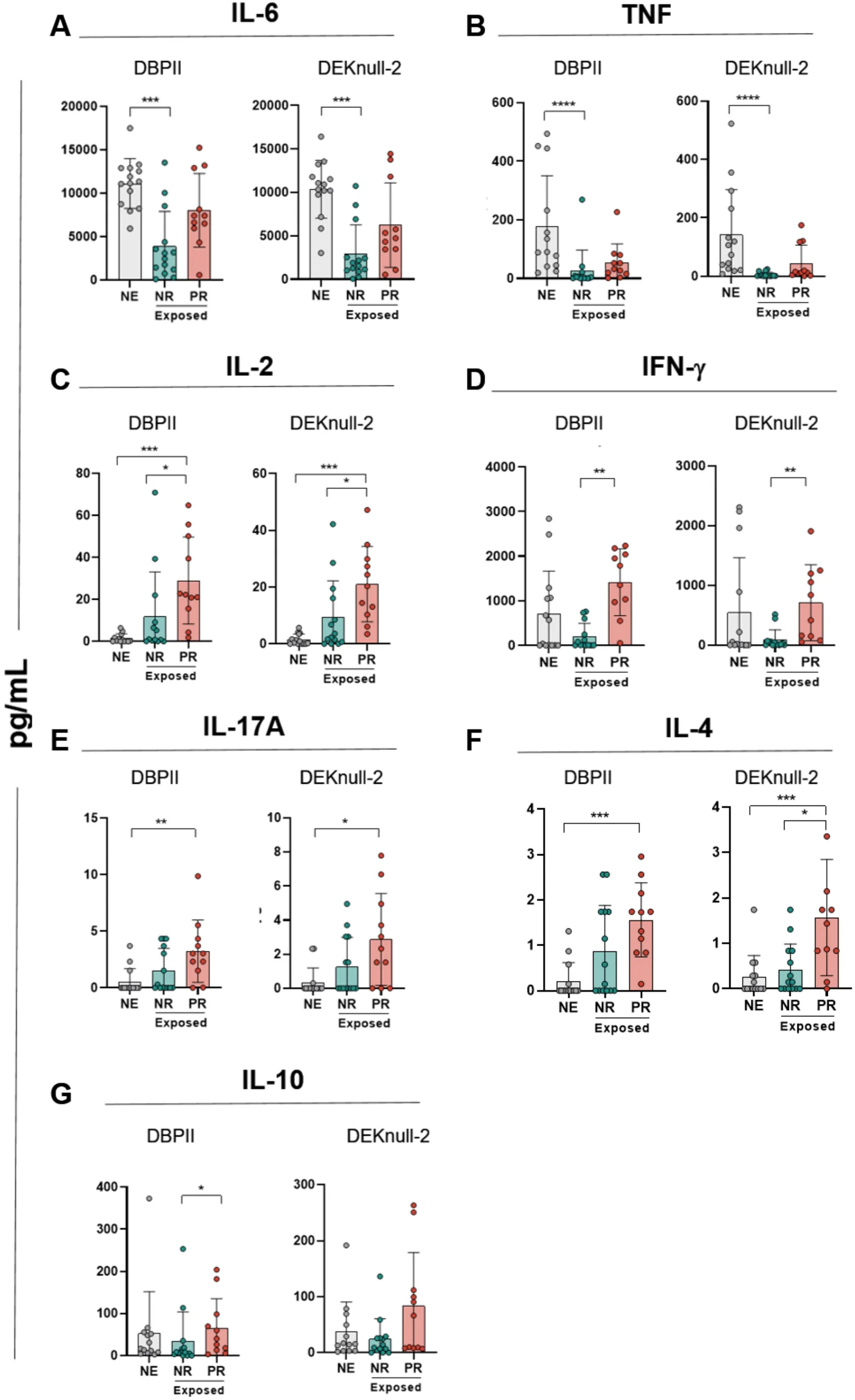
Cytokine concentrations after stimulation with DBPII or DEKnull-2. **(A–G)** Cytokine concentrations (pg/mL) in supernatants of antigen-stimulated PBMC cultures from non-endemic controls (NE, n = 14), non-responders (NR, n = 14), and persistent responders (PR, n = 11), measured using BD™ cytometric bead array (CBA) Human Th1/Th2/Th17 kit. Cytokines are presented individually: IL-6 **(A)**, TNF **(B)**, IFN-γ **(C)**, IL-2 **(D)**, IL-17A **(E)**, IL-4 **(F)**, and IL-10 **(G)**. Data are presented as individual values, with horizontal bars indicating the median and interquartile range. Statistical comparisons among NE, NR, and PR were performed using the Kruskal-Wallis test followed by Dunn’s multiple comparisons test and differences indicated by connecting lines and asterisks in the graphs (*P < 0.05; **P < 0.01; ***P < 0.001; ****P < 0.0001). Effect sizes, expressed as the Cliff’s delta values for all pairwise comparisons of cytokine production, indicated substantial differences between the groups. Cliff’s delta effect sizes with 95% bootstrap confidence intervals for all pairwise comparisons, as well as the comparisons that remained significant after additional Bonferroni correction, are provided in **Supplementary Table 4**. Raw cytokine concentrations measured in culture supernatants from unstimulated and antigen-stimulated cultures are provided in **Supplementary Table 5**.

Cytokine concentrations were measured in supernatants of PBMC cultures from malaria-exposed (PR and NR) and non-exposed (NE) individuals stimulated with DBPII or DEKnull-2 (**Figure 2**). Regardless of the antigen used for stimulation, malaria-exposed individuals (NR and PR) exhibited lower levels of IL-6 and TNF compared with non-exposed individuals (NE) (**Figures 2A, B**). No significant differences in these cytokines were observed between PR and NR subgroups. In contrast, PR individuals exhibited significantly higher levels of IL-2, IFN-γ, and IL-17A when compared with NR and NE subgroups (**Figures 2C–E**). In addition, PR individuals displayed increased levels of IL-4 and IL-10 after antigen stimulation (**Figures 2F, G**). Effect size, expressed as the Cliff’s delta values for all pairwise comparisons of cytokine production, indicated substantial difference between groups (**Supplementary Table 4**). The results showed that most of the comparisons remained statistically significant after Bonferroni correction, with 6 of 11 comparisons (55%) for DBPII and 8 of 11 comparisons (73%) for DEKnull-2 remaining significant (**Supplementary Table 4**). Together, this cytokine panel revealed quantitative differences in cytokine production across all subgroups. Additionally, cytokine production in unstimulated PBMC cultures revealed distinct baseline immune activation profiles (**Supplementary Table 5**), with the most pronounced differences observed between PR and NE individuals (**Supplementary Figure 3**).

### 3.2 Multivariate analysis of cytokine responses reveals differences among study subgroups

**Figure 3:**
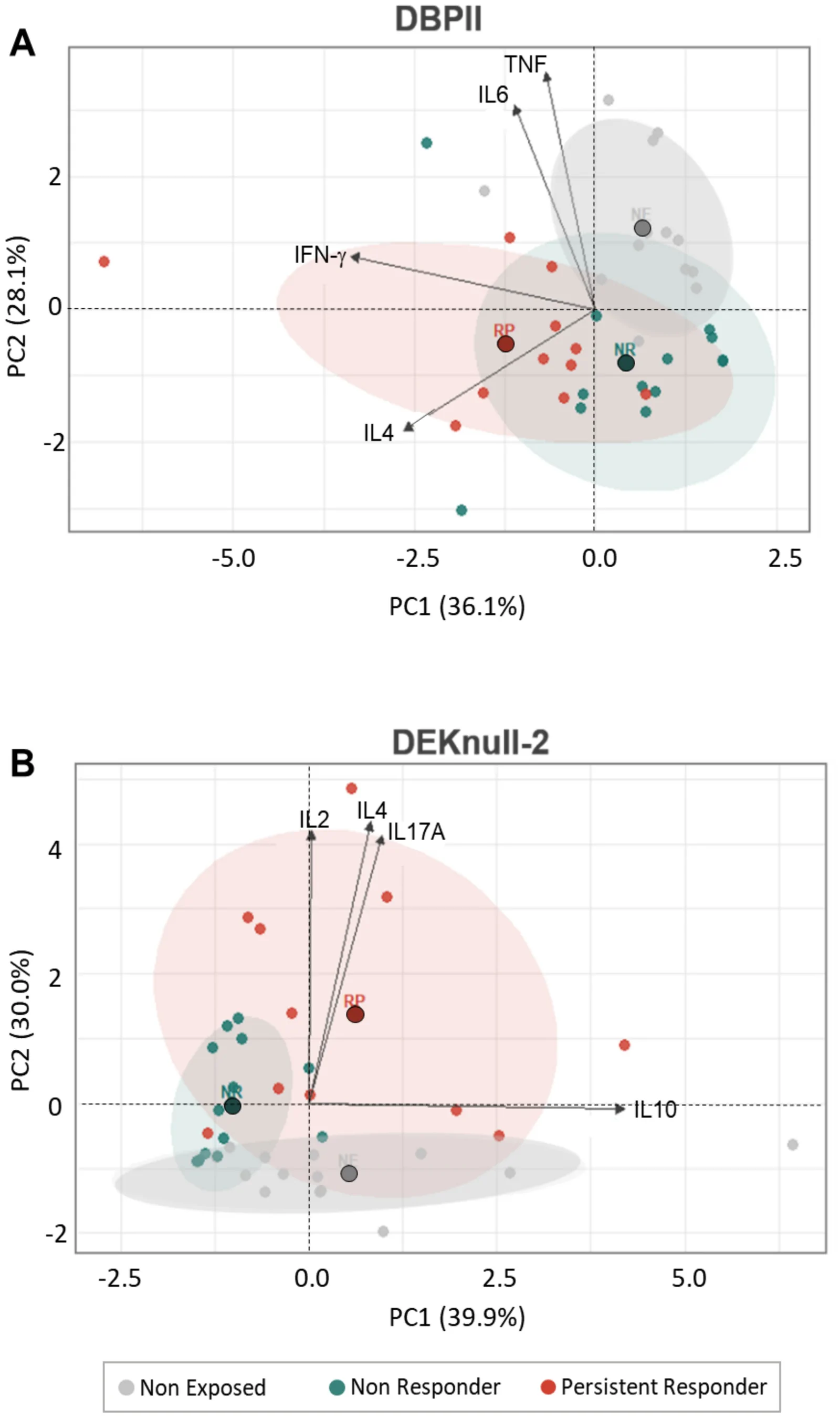
Multivariate analysis of cytokine responses after stimulation with DBPII and DEKnull-2. Principal component analysis (PCA) was performed using cytokine concentrations measured after *in vitro* stimulation of PBMCs with either **(A)** DBPII (Sal1) or **(B)** DEKnull-2. The plots display the distribution of non-exposed individuals (NE, n = 14) and malaria-exposed individuals classified as either non-responders (NR, n = 14) or persistent responders (PR, n = 11) along the first two principal components (PC1 and PC2), with the percentage of explained variance indicated on each axis. Each point represents an individual, and ellipses indicate group dispersion. Arrows represent cytokine loadings, indicating the direction and magnitude of the contribution of each cytokine to the principal components and the overall variance in the immune response pattern.

To determine whether differences observed at the individual cytokine level reflect coordinated immune response patterns, principal component analysis (PCA) was performed using combined cytokine data for each antigen stimulus (**Figures 3A, B**).

Following DBPII stimulation, the first two principal components explained 64.2% of the total variance (PC1 = 36.1% and PC2 = 28.1%) (**Figure 3A**), allowing visualization of the overall organization of cytokine responses among the subgroups of responders. The PR pattern was clearly distinct from NE, whereas NR exhibited an intermediate profile with partial overlap between subgroups. IFN-γ and IL-4 contributed predominantly to PC1, driving the separation of PR, while IL-6 and TNF loaded on PC2 and were associated with the NE profile.

After DEKnull-2 stimulation, PC1 and PC2 explained 69.9% of the total variance (PC1 = 39.9% and PC2 = 30.0%) (**Figure 3B**). PR subgroup formed a broader and more distinct cluster, whereas NR and NE subgroups remained closely distributed. IL-2, IL-4, and IL-17A mainly loaded on PC2, associated with the PR pattern, whereas IL-10 predominantly loaded on PC1, reflecting variability within PR subgroup. Overall, PCA demonstrated that cytokine responses are organized into distinct multivariate patterns, with segregation observed among PR individuals.

### 3.3 Cytokine landscape associated with persistent antibody responses

**Figure 4:**
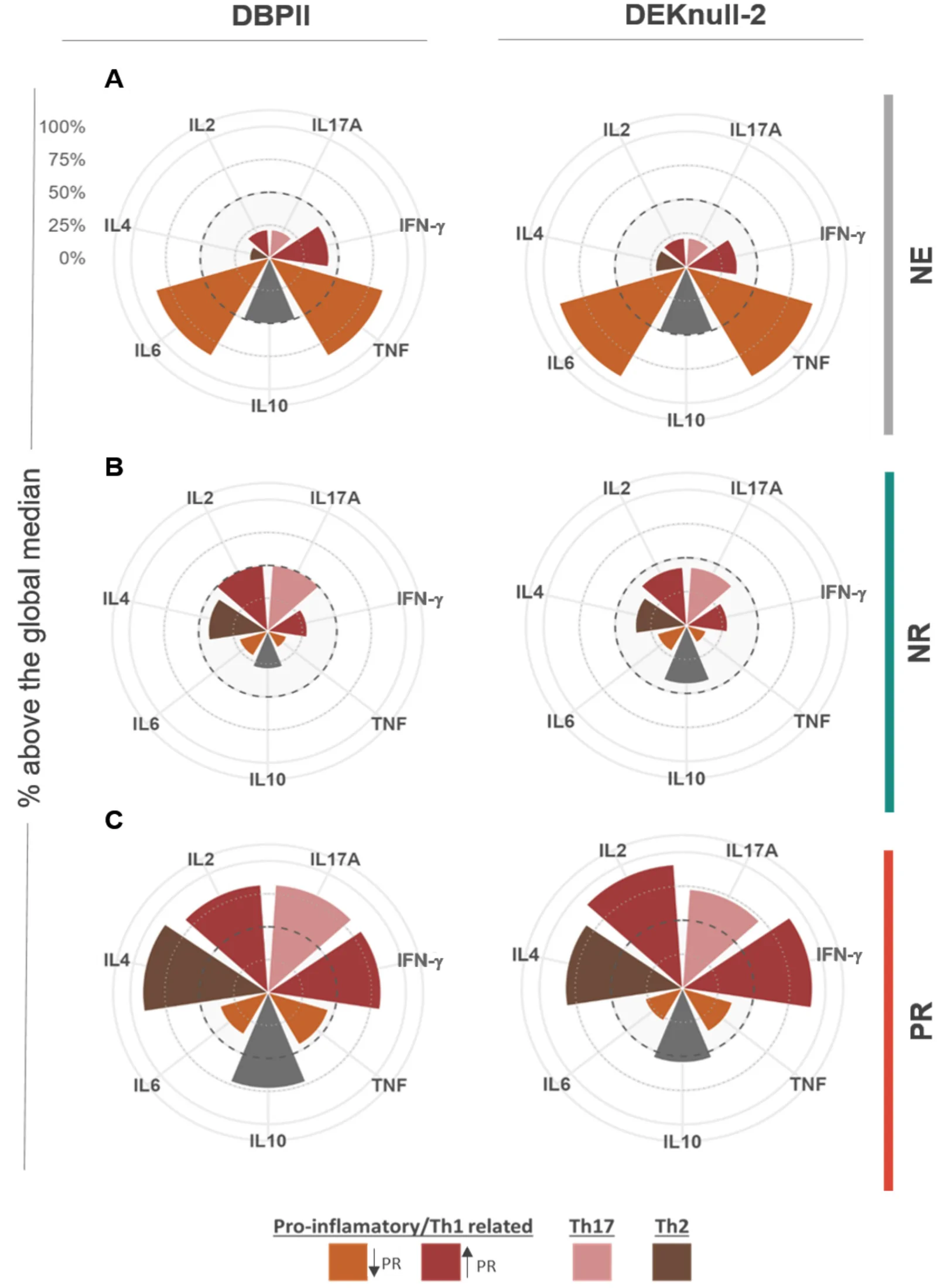
Radial signature cytokine analysis for DBPII and DEKnull-2 in the study subgroups. Wheel plots providing descriptive overviews of the proportion of individuals exhibiting cytokine concentrations above the global median for each cytokine after *in vitro* stimulation of PBMCs with either DBPII-Sal1 (left panel) or DEKnull-2 (right panel), stratified by study subgroups: **(A, B)** malaria non-exposed individuals residing in a non-endemic area (NE, n = 14), **(C, D)** persistent non-responders (NR, n = 14), and **(E, F)** persistent responders (PR, n = 11). Larger polygon areas indicate broader coordinated cytokine responses across the evaluated immune profiles. For each cytokine, the global median was calculated using all participants within each stimulus condition and was subsequently used as a common threshold to classify each individual as above or below the median. Each axis corresponds to a measured cytokine (IL-2, IL-17A, IFN-γ, TNF, IL-6, IL-10, and IL-4), and radial values ​​indicate the percentage of individuals above the global median for each cytokine. For descriptive purposes, cytokines were color-coded according to their predominant immunological functions, following the manufacturer’s classification (Human Th1/Th2/Th17 Cytokine CBA kit), and their predominance between sub-groups: pro-inflammatory/Th1 associated with NE (IL-6, TNF), pro-inflammatory/Th1 associated with PR (IFN-γ, IL-2), Th17 (IL-17A), and Th2 (IL-4, IL-10).

To further characterize functional differences suggested by the PCA, cytokine responses to DBPII and DEKnull-2 were represented using radial signature analysis, considering the proportion of individuals with cytokine levels above the global median for each stimulus across all subgroups (**Figure 4**). As compared with malaria-exposed individuals (NR and PR), non-exposed individuals (NE) displayed a distinct profile dominated by pro-inflammatory responses, with a higher proportion of individuals exhibiting higher levels of IL-6 and TNF following stimulation with either DBPII or DEKnull-2 (**Figures 4A, B**). In contrast, NR and PR showed a lower proportion of individuals with elevated IL-6 and TNF for both antigens (**Figures 4C–F**). In general, NR and PR showed similar cytokine profiles differing mainly in the magnitude of the response (**Figures 4C–F**). Of interest, the PR subgroup had more individuals above the global median across multiple immunological axes, including pro-inflammatory/Th1-associated cytokines (IL-2 and IFN-γ), a Th17-associated cytokine (IL-17A), and Th2-associated cytokines (IL-4 and IL-10) (**Figures 4E, F**).

### 3.4 Immune profile of persistent responders to multiple *P. vivax* blood-stage antigens

**Figure 5:**
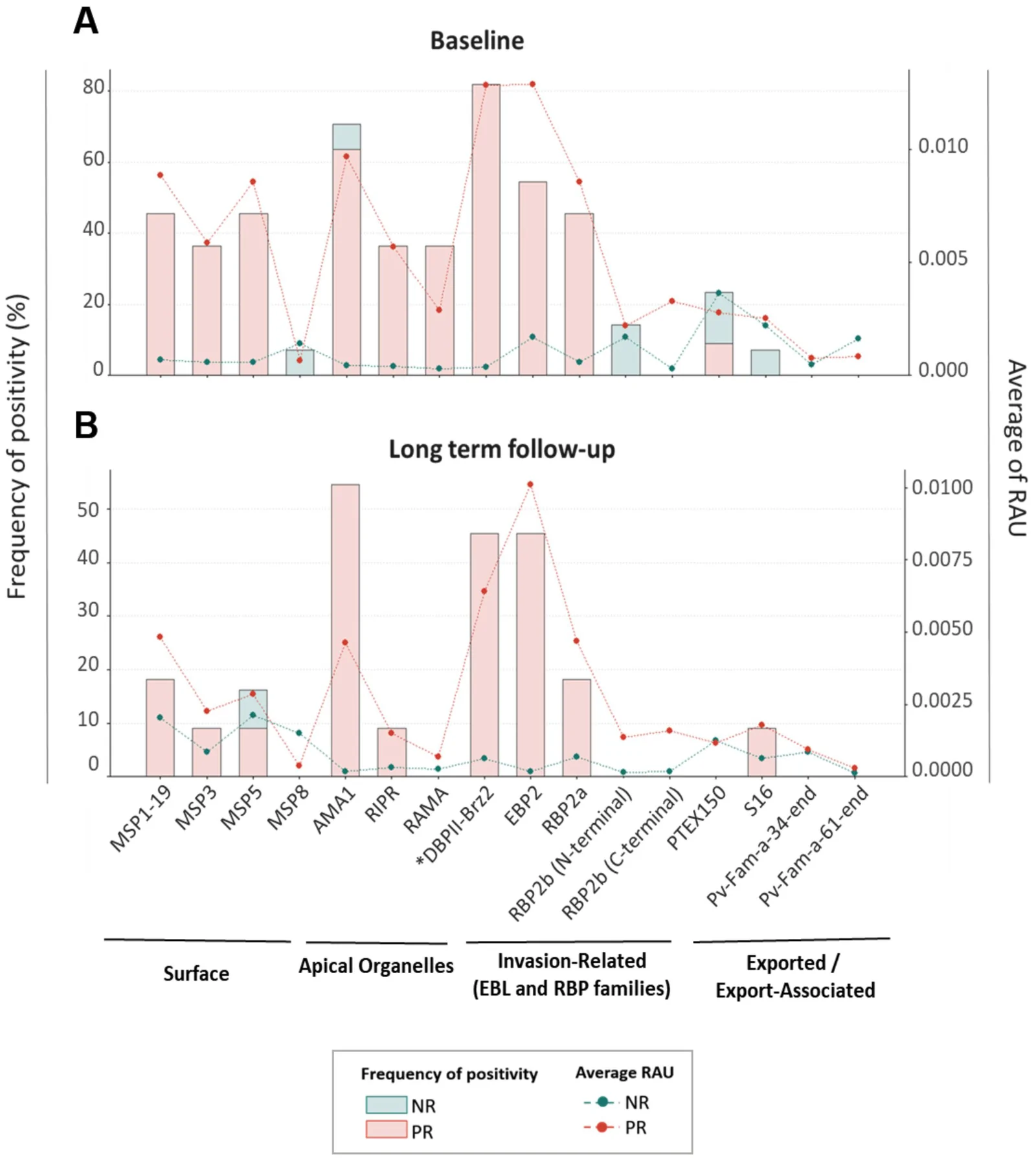
Frequency and magnitude of IgG responses to *Plasmodium vivax* blood-stage antigens. The graphs show the frequency of seropositive individuals (%) for each antigen (bars, left y-axis) and the magnitude of the IgG response, expressed as relative antibody units (RAU) (dashed lines, right y-axis), at baseline **(A)** and during long-term follow-up **(B)**. Persistent non-responders (NR, n = 14; light green) and persistent responders (PR, n = 11; red) are shown. Seropositivity frequencies were calculated using previously established antigen-specific cut-offs. Differences in IgG response magnitude between PR and NR were evaluated for each antigen and showed large effect sizes at both baseline and long-term follow-up (Cliff’s |δ| = 0.51–0.96). Exact *P* values, Cliff’s delta effect sizes, and comparisons remaining significant after additional Bonferroni correction are provided in **Supplementary Table 7**.

To determine whether the Persistent Responder (PR) phenotype was associated with broader and long-lasting humoral responses beyond DBPII-related antigens, antigen-specific IgG responses to a panel of 16 P*. vivax* blood-stage antigens were measured using the Bio-Plex platform in malaria-exposed individuals (PR and NR) at baseline and after 10 years of follow-up (**Figure 5**). At baseline, PR individuals exhibited IgG responses to multiple *P. vivax* blood-stage antigens, with prominent reactivity to surface antigens (MSP1-19 and MSP5), the apical antigen AMA1, and invasion-related antigens, including RBP2a, DBPII-Brz2, and EBP2. For several antigens, PR individuals showed both higher frequency of seropositivity and increased levels of relative antibody units (RAU) compared with NR individuals (**Figure 5A**).

Longitudinal analysis showed that the antibody profile observed at baseline was maintained in PR individuals after the long-term follow-up period (**Figure 5B**), with no significant decline in RAU levels (**Supplementary Figure 4**). Antigens with higher seropositivity frequencies at baseline remained the most prevalent over time, with sustained responses observed for AMA1, DBPII-Brz2, and EBP2. Differences in antibody response levels between PR and NR individuals were further supported by large effect sizes at both baseline and follow-up (Cliff’s delta, |δ| = 0.51–0.96; **Supplementary Table 7**). Following Bonferroni correction for multiple comparisons, most of the significant differences between groups remained statistically significant at both time points. Specifically, 8 of 13 comparisons (62%) at baseline and 7 of 11 comparisons (64%) at follow-up remained significant. Taken together, these findings demonstrate that Persistent Responders develop a broad and stable humoral immune response extending beyond DBPII-Sal1 and DEKnull-2, including multiple *P. vivax* blood-stage vaccine candidates.

## 4 Discussion

There seems to be a consensus that immunity to clinical *P. vivax* infection is acquired more quickly than to *P. falciparum*, and that individuals in endemic areas appear to develop stronger immunological memory (39–41). However, the mechanisms sustaining *P. vivax* long-term immunity remain poorly understood. Therefore, identifying the immune features associated with persistent antibody responses may help address this gap in our knowledge of malaria infection.

In this study, it is demonstrated that long-term *P. vivax*-exposed Amazonian individuals, who have acquired strain-transcending immune response to DBPII (persistent responders, PR), exhibited a distinct immunological profile characterized by regulation and expansion of multiple functional cytokine pathways. Compared with non-exposed individuals (NE), PR showed reduced levels of inflammatory cytokines IL-6 and TNF, key mediators of systemic inflammation and disease severity in malaria (42–44), alongside increased levels of IFN-γ, IL-2, IL-4, IL-17A, and IL-10. Although non-responder *P. vivax*-exposed (NR) individuals also showed reduced IL-6 and TNF levels, this was not accompanied by a coordinated expansion of other functional cytokine pathways, suggesting that modulation of inflammatory cytokine responses is necessary, but not sufficient to sustain long-term humoral immunity.

In our study population, where individuals had a history of long-term exposure to low levels of malaria transmission (28, 29), the existence of a subgroup of non-responders is noteworthy. Although the reasons why only a subset of exposed individuals develop long-lasting anti-DBPII antibody responses remain incompletely understood, host genetic factors may contribute to this variability. Indeed, we have previously shown that, in the Brazilian Amazon, both HLA class II alleles (45) and DARC genotypes (46) are associated with the development of anti-DBPII antibody response. Notably, despite substantial epidemiological and host–parasite genetic differences between the Brazilian Amazon and highly endemic areas such as Papua New Guinea (PNG), studies from both settings consistently show that only a relatively small proportion of individuals develop DBPII-binding inhibitory antibodies (BIAbs) [reviewed by de Souza et al., 2014 (47)]. More in-depth studies are warranted to elucidate why some exposed individuals fail to develop antibody responses to DBPII-related antigens.

Considering that DBPII induces strain-specific immune responses (12, 48), it was important to investigate whether cytokine response patterns following stimulation with DBPII-related antigens could be associated with either strain-specific (Sal1, a natural DBPII variant circulating in the study area) or strain-transcending immune responses (DEKnull-2, conserved epitopes). Although PCA showed some discrete differences between the antigens, overall, the response profile was similar for both DBPII and DEKnull-2. This pattern was not unexpected as persistent responders are associated with a stable antibody response against multiple DBPII variants (18). In fact, the overall response landscape became evident through radial cytokine signature analysis, which revealed that, regardless of antigen stimulation, the PR subgroup was characterized by a high frequency of individuals with elevated levels of multiple cytokines. In contrast, NR and NE subgroups lacked this multi-axis cytokine elevation. In line with this immunological landscape, pivotal pro-inflammatory cytokines show inverse regulation patterns in the PR subgroup, with upregulation of IFN-γ associated with reduced TNF and IL-6 responses, suggesting a balance between protective immune responses and immunopathology (49). Although limited information is still available on the role of these mediators in the long-term protective humoral immune response to *P. vivax*, recent studies support our findings by suggesting a critical role for the IFN-γ-driven immunity of individuals with asymptomatic *P. vivax* infection (31, 50). At present, there is an ongoing debate regarding the B- and T-cell subsets associated with IFN-γ signaling that may contribute to distinct infection outcomes (31, 50–52).

Unlike asymptomatic *P. vivax* infections, which are typically characterized by submicroscopic parasitemia (53), participants in our cohort classified as persistent responders (PR) did not present circulating parasites, as confirmed by consecutive PCR-based protocols over time (**Supplementary Table 3**). These differences may contribute to the divergent cytokine profiles observed between this cohort and previous studies of asymptomatic *P. vivax* individuals, in which cytokines such as IL-4 were reported to be decreased (31). Here, PR presented high levels of both IL-4 and IFN-γ as compared with other groups, consistent with previous studies indicating that these cytokines are strong predictors of the protective immunity induced by attenuated malaria vaccines (54, 55). Further studies should investigate the complex nature of cellular interactions required for long-term DBPII protective immune responses.

The PR signature described here also included elevated levels of IL-17A, a member of the IL-17 family, primarily associated with Th17 cells and inflammatory response (56). Recent evidence has identified a subset of follicular helper T cells (Tfh17) that retains canonical B-cell helper functions while producing IL-17 [reviewed by Shen and Fu et al., 2025 (57)]. Consistent with this, Th17 cells have been associated with protection against asymptomatic *P. vivax* infection (50). Collectively, these findings support further mechanistic studies to clarify the role of the IL-17 pathway in protective immune responses against *P. vivax*.

Considering that antibodies against a range of *P. vivax* blood-stage antigens have been associated with protection (3, 20, 58), the breadth of humoral responses was further investigated within the PR subgroup. These individuals exhibited a broad antibody reactivity, characterized by higher frequencies and levels of antibodies across multiple *P*. *vivax* antigens. This was particularly noteworthy, as these responses targeted proteins with diverse parasite localizations and functions, including merozoite surface proteins, exported/associated proteins, apical organelle proteins, and different families of invasion-related proteins. Notably, antibody responses to key blood-stage antigens, including AMA1, and EBP2, remained relatively stable for up to 15 years in the absence of frequent reinfection-induced boosting. This pattern of long-lasting humoral immunity is consistent with previous findings in malaria-exposed individuals, in whom antigen-specific antibodies can be maintained for prolonged periods in the absence of reinfection, potentially through mechanisms involving long-lived plasma cells and/or memory B cells (59, 60). At this point, the contribution of cryptic *P. vivax* infections to the maintenance of these long-lived antibody responses cannot be excluded, as parasites may persist in hidden niches such as the bone marrow and spleen (61, 62) potentially sustaining low-level antigenic exposure. This is particularly relevant as this cohort consisted mainly of semi-immune adults (median age, 36 years; IQR, 18–51), who were likely exposed to subpatent *P. vivax* infections that occurred between sampling visits and/or remained undetected because they were below the detection limit of the PCR protocols used (32), consistent with cryptic *P. vivax* infections. Thus, future studies should investigate whether *P. vivax* IgG-experienced B cells need to be constantly activated or if this response is rather associated with a bona-fide memory (27).

The current study has limitations that should be considered when interpreting the data. The convenience sampling approach and the small sample size may have limited the statistical power and precluded more robust statistical analyses. Nevertheless, Bonferroni correction for multiple comparisons confirmed the robustness of the main findings, with most differences between groups remaining statistically significant. Indeed, the inclusion of all participants who met the predefined eligibility criteria and had available biological samples reduced the risk of bias. Accordingly, selection bias due to the exclusion of non-eligible participants was unlikely to have substantially affected the results, as the retained participants were comparable to the original cohort with respect to demographic, epidemiological, and parasitological characteristics, making it unlikely that confounding variables influenced the findings. In addition, effect sizes estimated by using Cliff’s delta (38) consistently indicated substantial differences between study groups. Finally, as a proof-of-concept study, this work compared two well-defined immunological phenotypes (PR vs. NR) rather than estimating population-level parameters. Consequently, residual confounding due to unmeasured factors, including host genetics, variability in malaria exposure, cryptic infections, and other environmental and behavioral factors, cannot be completely excluded. Nevertheless, the cytokine findings should be interpreted as descriptive and hypothesis-generating, and the conclusions should be framed as associative rather than causal, given the small sample size and the absence of cellular phenotyping.

Although the recombinant proteins were purified according to the Standard Operating Procedures (SOPs) of the Recombinant Protein Facility (PROREC) at Fiocruz Minas, the potential presence of residual *E. coli*-derived components, such as LPS, cannot be completely excluded and should be considered when interpreting cytokine responses following PBMC stimulation. Despite that, residual endotoxin alone is unlikely to explain the cytokine profiles as: (i) all study groups were stimulated under identical experimental conditions, such that any residual endotoxin would be expected to affect cytokine responses similarly across groups; and (ii) PR showed lower levels of IL-6 and TNF, despite these cytokines typically being elevated during acute TLR4-mediated endotoxin stimulation (63). Considering antigen-specific responses, it is also worth emphasizing that differences observed among groups in unstimulated cultures suggest distinct baseline immune states. Although the reasons for this require further investigation, these findings are consistent with recent evidence suggesting that pre-existing immune status may shape vaccine-induced immunity (54, 64, 65). Supporting the role of host-intrinsic factors in maintenance of long-term humoral immunity, we previously demonstrated that specific HLA class II alleles influenced the development and persistence of anti-DBPII antibody responses (45). Thus, host-intrinsic factors should be considered when interpreting antigen-specific immune responses.

Finally, the experimental approach focused on characterizing distinct cytokine profiles rather than identifying the cellular sources of these cytokines or defining specific signaling pathways associated with these responses. Consequently, the absence of cellular phenotyping limited the identification of the primary cellular sources of cytokines associated with the PR profile. Ongoing experiments are in progress to identify T and B cell subsets associated with this long-lived antibody response. This is particularly relevant, as evidence from acute and convalescent *P. vivax* infections suggests that IFN-γ promotes the differentiation of atypical memory B cells capable of generating antibodies that inhibit the DBPII-DARC interaction (52, 66).

Overall, our findings reported here indicate that individuals with persistent strain-transcending DBPII immune responses displayed a distinct cytokine profile compared with non-responders from the same endemic area. However, cytokine findings are descriptive and hypothesis-generating, given the small sample size and lack of cellular phenotyping. Further characterization of the immune mechanisms underlying these cytokine patterns may provide insights into vaccine strategies targeting *P. vivax* blood-stage antigens.

## Data Availability

The original contributions presented in the study are included in the article/**Supplementary Material**. Further inquiries can be directed to the corresponding authors.
