# Supplementary material for: Cytokine signatures associated with persistent antibody responses to multiple Plasmodium vivax blood-stage antigens
Source: Front Immunol. 2026 Aug 31;17:1899417. doi: 10.3389/fimmu.2026.1899417 (PMC13572285; doi:10.3389/fimmu.2026.1899417)
Supplement: Supplementary file 1 [file DataSheet1.pdf]

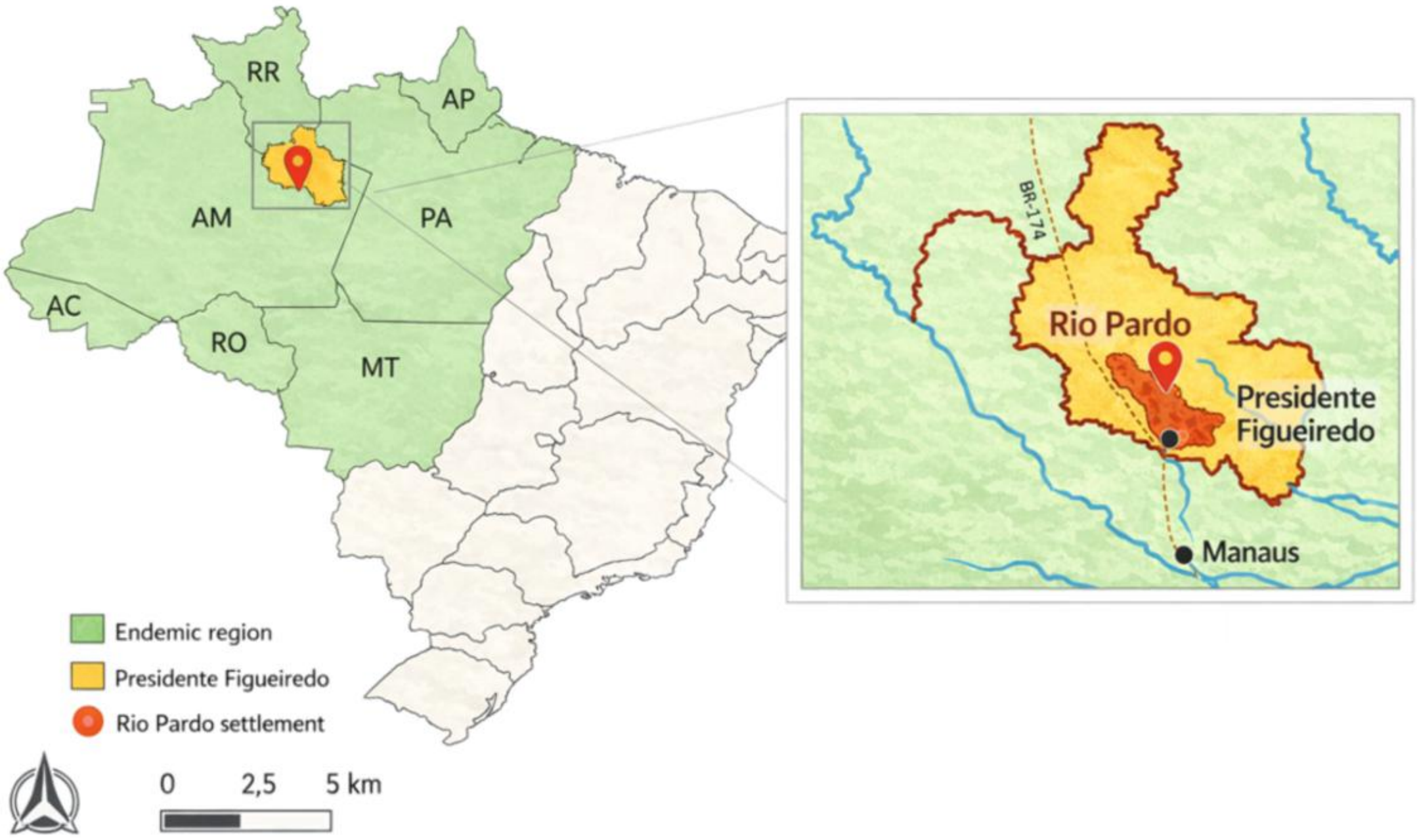

Supplementary Figure 1. Geographic location of the study site. Map of Brazil, highlighting the malaria-endemic region in the Brazilian Amazon (green) and, the location of the Rio Pardo agricultural settlement (red), municipality of Presidente Figueiredo (orange), state of Amazonas. Figure adapted from Kano et al., (2012) and refined using generative artificial intelligence tools.
