# Supplementary material for: Cytokine signatures associated with persistent antibody responses to multiple Plasmodium vivax blood-stage antigens
Source: Front Immunol. 2026 Aug 31;17:1899417. doi: 10.3389/fimmu.2026.1899417 (PMC13572285; doi:10.3389/fimmu.2026.1899417)
Supplement: Supplementary file 2 [file DataSheet2.pdf]

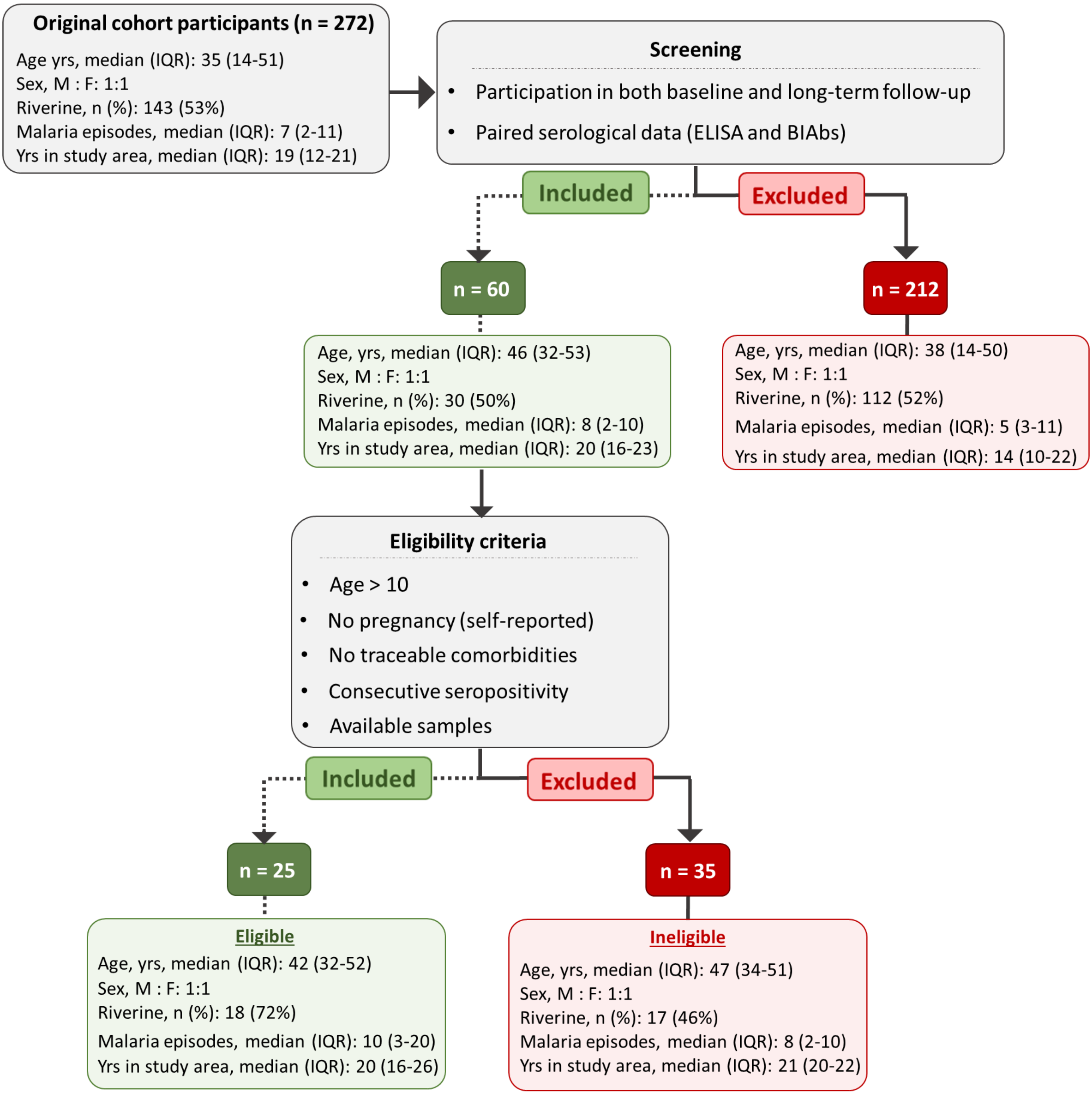

Supplementary Figure 2. Flow diagram of participant selection and eligibility throughout the study. The diagram summarizes the selection process from the original population-based cohort (n = 272) to the final study population (n = 25). No statistically significant differences were detected in the demographic, parasitological, or epidemiological characteristics between study participants retained in the cohort and those excluded (Suppl. Table 1).
