# Supplementary material for: Cytokine signatures associated with persistent antibody responses to multiple Plasmodium vivax blood-stage antigens
Source: Front Immunol. 2026 Aug 31;17:1899417. doi: 10.3389/fimmu.2026.1899417 (PMC13572285; doi:10.3389/fimmu.2026.1899417)
Supplement: Supplementary file 3 [file DataSheet3.pdf]

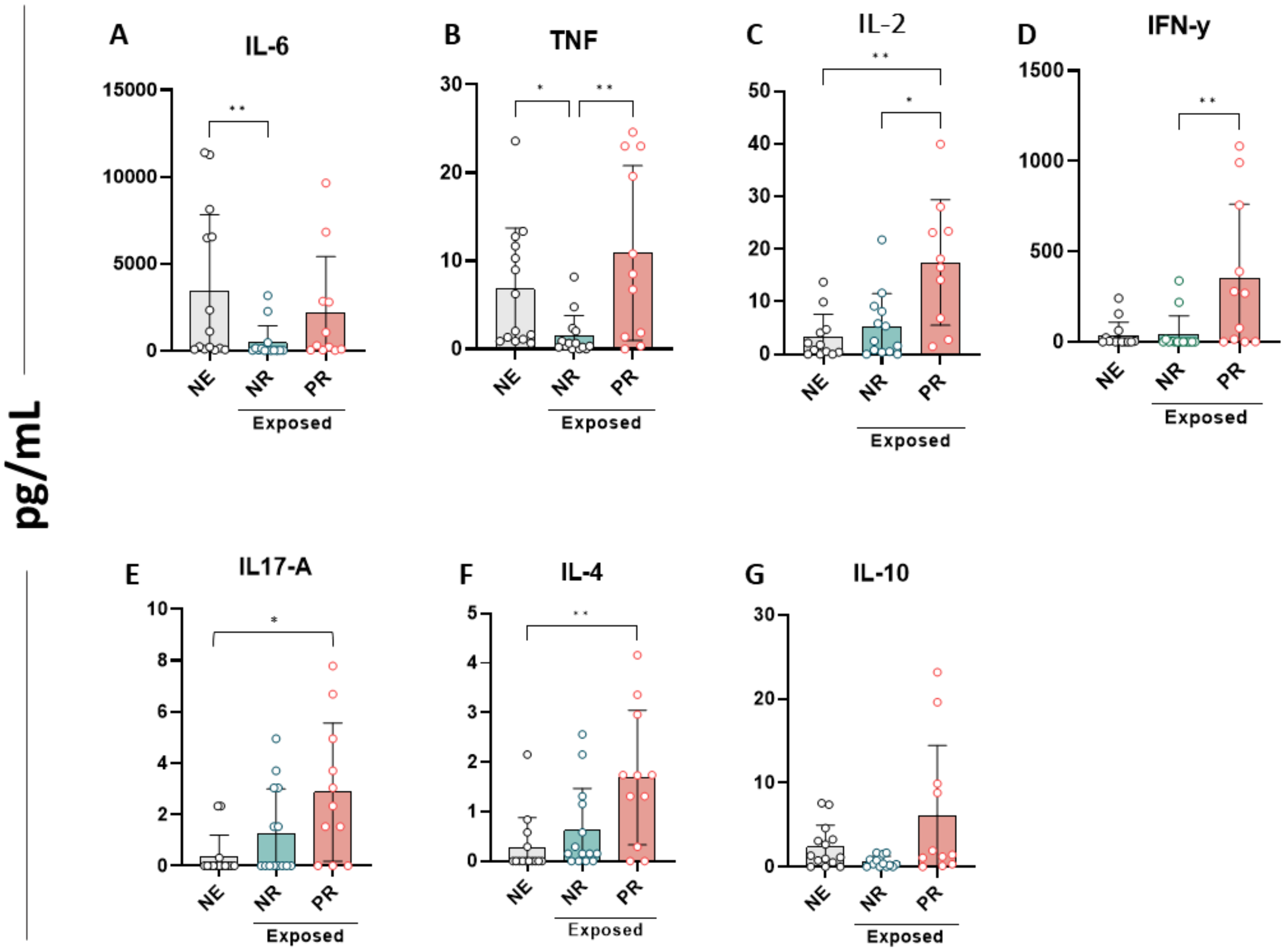

Supplementary Figure 3. Cytokine concentrations in unstimulated PBMC cultures. (A–G) Cytokine concentrations (pg/mL) measured in supernatants from unstimulated PBMC cultures of non-endemic controls (NE), non-responders (NR), and persistent responders (PR), using the BD™ Cytometric Bead Array (CBA) Human Th1/Th2/Th17 Cytokine Kit. Cytokines are shown individually: IL-6 **(A)**, TNF **(B)**, IFN-γ **(C)**, IL-2 **(D)**, IL-17A **(E)**, IL-4 **(F)**, and IL-10 **(G)**. Data are presented as individual values, with bars indicating the median and interquartile range. Statistical comparisons among groups were performed using the Kruskal–Wallis test followed by Dunn's multiple-comparisons test. Significant differences are indicated by connecting lines and asterisks: **P* < 0.05; ***P* < 0.01; ****P* < 0.001; *****P* < 0.0001. Effect size, expressed as the Cliff's delta values for all pairwise comparisons of cytokine production, indicated substantial difference between groups (Suppl. Table 6).
