# Supplementary material for: Cytokine signatures associated with persistent antibody responses to multiple Plasmodium vivax blood-stage antigens
Source: Front Immunol. 2026 Aug 31;17:1899417. doi: 10.3389/fimmu.2026.1899417 (PMC13572285; doi:10.3389/fimmu.2026.1899417)
Supplement: Supplementary file 4 [file DataSheet4.pdf]

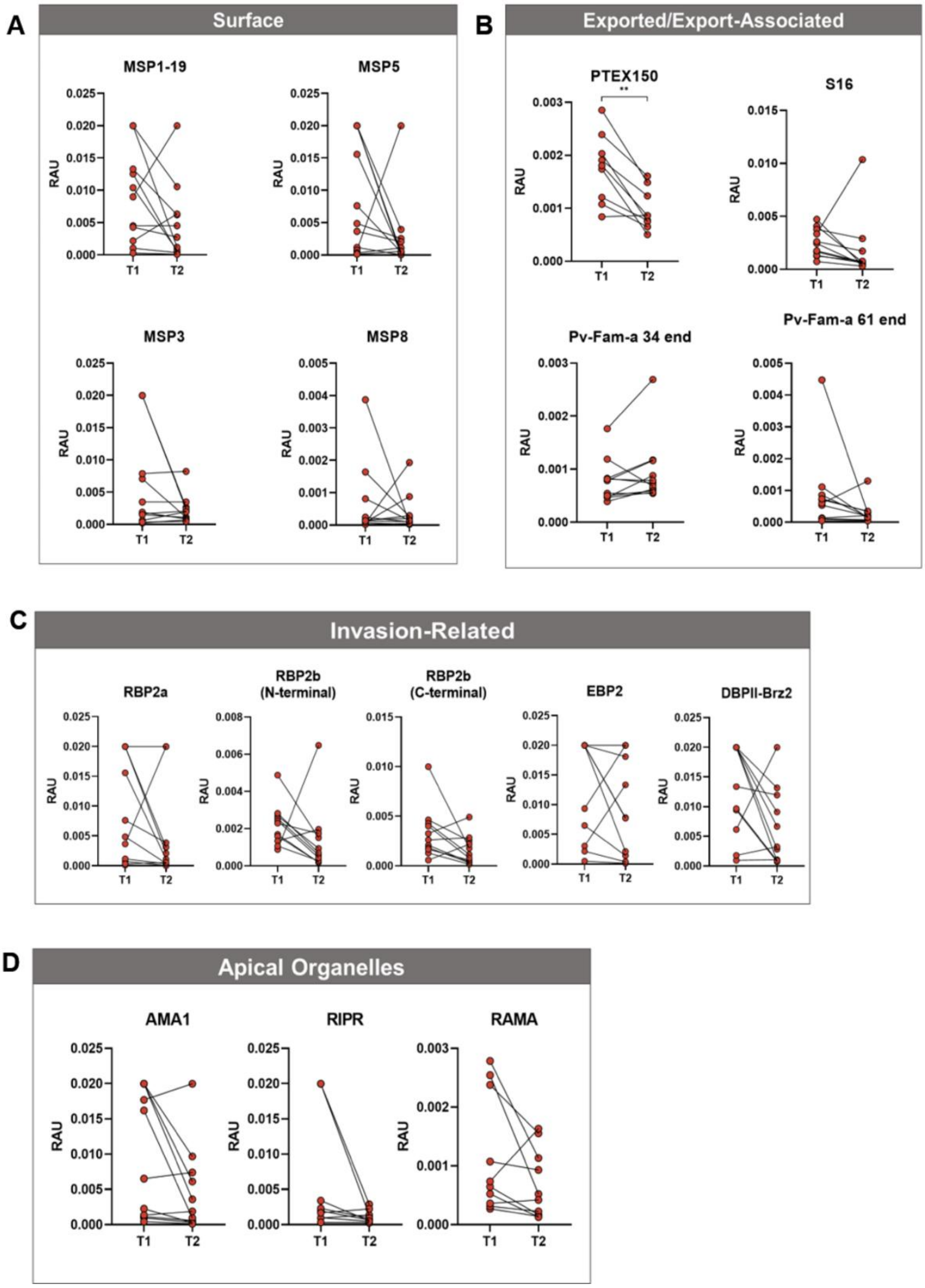


**Supplementary Figure 4. Intensity of IgG responses to *Plasmodium vivax* blood stage antigens in persistent responders (PR) at baseline (T1) and long-term follow-up (T2).** The intensity of the IgG response, expressed as relative antibody units (RAU), is shown for individual *P. vivax* blood-stage antigens in persistent responders (PR) in baseline and approximately 15 years later (long-term follow-up). Antigens are grouped according to their location or function, including **(A)** surface proteins, **(B)** exported or export-associated proteins, **(C)** invasion-related antigens, and **(D)** apical organelles. Each point represents an individual, and lines connect the responses of the same individual between the two periods evaluated.
