# Supplementary material for: Cytokine signatures associated with persistent antibody responses to multiple Plasmodium vivax blood-stage antigens
Source: Front Immunol. 2026 Aug 31;17:1899417. doi: 10.3389/fimmu.2026.1899417 (PMC13572285; doi:10.3389/fimmu.2026.1899417)
Supplement: Supplementary file 5 [file DataSheet5.pdf]

SUPPLEMENTARY DATA

Standard Operating Procedure (SOP): In Vitro Antigen-Specific Stimulation of Human PBMCs for Cytokine Profiling

Purpose: This Standard Operating Procedure (SOP) describes the methodology for in vitro stimulation of cryopreserved human peripheral blood mononuclear cells (PBMCs) with recombinant *Plasmodium vivax* Duffy Binding Protein region II (DBPII-Sal1) and DEKnull-2 antigens, followed by cytokine quantification in culture supernatants using Cytometric Bead Array (CBA).

Scope: This procedure was used to assess cytokine production after six days of PBMC culture under antigen-stimulated and control conditions.

MATERIALS

Reagents

- Cryopreserved human PBMCs
- CMBlast medium consisting of:
  - RPMI 1640
  - 5% heat-inactivated fetal bovine serum (FBS)
  - 3.2 mM L-glutamine
  - Penicillin (300 U/mL)
  - Streptomycin (300 µg/mL)
- Benzonase® nuclease (Sigma-Aldrich), 25 U/mL
- Recombinant DBPII-Sal1 antigen
- Recombinant DEKnull-2 antigen
- Sterile phosphate-buffered saline (PBS), if required
- BD™ Cytometric Bead Array (CBA) Human Th1/Th2/Th17 Kit (BD Biosciences; Cat. No. 560484)

Equipment

- 37°C water bath
- Refrigerated centrifuge
- Biological safety cabinet
- CO_2 incubator (37°C, humidified, 5% CO_2)
- Sterile 96-well flat-bottom tissue culture plates
- Micropipettes and sterile filtered tips
- BD FACSVerse™ flow cytometer
- FCAP Array v3.0 software

PROCEDURE

1. PBMC Thawing

1. Remove cryovials from liquid nitrogen storage.
2. Rapidly thaw PBMCs in a 37°C water bath until only a small ice crystal remains.
3. Immediately transfer the cell suspension to 10 mL of pre-warmed CMBlast medium supplemented with 25 U/mL Benzonase® nuclease.
4. Wash the cells according to standard laboratory procedures.
5. Resuspend PBMCs in fresh CMBlast medium.
6. Determine cell count and viability.
7. Only PBMC preparations exhibiting post-thaw viability greater than 80% should be used for subsequent culture.

2. Cell Culture and Antigen Stimulation

1. Prepare PBMC suspensions at a density sufficient to seed 5×10^5 cells per well in a final volume of 200 µL CMBlast medium.
2. Dispense cells into sterile 96-well round-bottom plates in a final volume of 200 µL per well.
3. Add recombinant DBPII-Sal1 or DEKnull-2 antigen from sterile stock solutions to achieve a final concentration of 5 µg/mL.
4. Prepare unstimulated control wells by adding an equivalent volume of culture medium without antigen.
5. Set up all culture conditions in duplicate.

3. Culture Conditions

1. Incubate cultures at 37°C in a humidified atmosphere containing 5% CO_2.
2. Maintain cultures for 6 days without disturbing the plates or replacing the medium.
3. The antigen concentration (5 µg/mL) and incubation period (6 days) were previously optimized to promote antigen-specific recall responses and cytokine production.

4. Collection of Culture Supernatants

1. At the end of the 6-day incubation period, centrifuge the plates at 800 × g for 5 min.
2. Carefully collect the culture supernatants without disturbing the cell pellet.
3. Process the supernatants immediately for cytokine quantification or store them at -80°C until analysis.

5. Cytokine Quantification

1. Quantify cytokines using the BD™ Cytometric Bead Array (CBA) Human Th1/Th2/Th17 Kit according to the manufacturer's instructions.

2. Acquire samples using a BD FACSVerse™ flow cytometer.
3. Analyze data using FCAP Array v3.0 software.

Quality Control

- Use only PBMC samples with post-thaw viability >80%.
- Prepare complete culture medium using sterile technique.
- Include unstimulated-control wells for each donor.
- Perform all experimental conditions in duplicate.
- Maintain constant incubation conditions (37°C, humidified atmosphere, 5% CO_2) throughout the culture period.

Critical Notes

- Based on previous studies (1, 2), two different antigen concentrations were evaluated (5 µg/mL and 10 µg/mL), with 5 µg /mL yielding the best results.
- The incubation time was optimized by testing different stimulation durations (one, three and six days) with 6-day incubation period providing to the most appropriate for cytokine secretion.

References

1. Changrob S, Han JH, Ha KS, Park WS, Hong SH, Chootong P, et al. Immunogenicity of glycosylphosphatidylinositol-anchored micronemal antigen in natural *Plasmodium vivax* exposure. Malar J. 2017;16(1):348. doi:10.1186/s12936-017-1967-9 PubMed PMID: 28830553.

2. Thawornpan P, Changrob S, Kochayoo P, Wangriatisak K, Ntumngia FB, De SL, et al. Correction: Cross-reactive inhibitory antibody and memory B cell responses to variant strains of Duffy binding protein II at post-*Plasmodium vivax* infection. PLoS One. 2025;20(3):e0320801. doi:10.1371/journal.pone.0320801 PubMed PMID: 40095987.
